# Comparative genomics of Pantoea allii lineages and distribution of ecologically relevant traits

**DOI:** 10.1099/mgen.0.001624

**Published:** 2026-02-04

**Authors:** Gi Yoon Shin, Stefanie De Armas, Guillermo A. Galván, María I. Siri, Mariah Rojas, Boris A. Vinatzer, Jo Ann E. Asselin, Paul Stodghill, Mei Zhao, Bhabesh Dutta, James Tambong, Brian H. Kvitko

**Affiliations:** 1 Department of Plant Pathology, University of California Davis, Davis, CA, USA; 2 Laboratorio de Microbiología Molecular, Facultad de Química, Universidad de la República, Montevideo, Uruguay; 3 Departamento de Producción Vegetal (CRS), Facultad de Agronomía, Universidad de la República, Montevideo, Uruguay; 4 School of Plant and Environmental Sciences, Virginia Tech, Blacksburg, VA, USA; 5 Emerging Pests and Pathogens Research Unit, Robert W. Holley Center for Agriculture and Health, Agricultural Research Service, United States Department of Agriculture, Ithaca, NY, USA; 6 Plant Pathology & Plant-Microbe Biology Section, School of Integrated Plant Science, Cornell University, Ithaca, NY, USA; 7 Department of Plant Pathology, College of Plant Protection, China Agricultural University, Beijing, PR China; 8 Department of Plant Pathology, University of Georgia, Tifton, GA, USA; 9 Ottawa Research and Development Centre, Agriculture and Agri-Food Canada, Ottawa, ON, Canada; 10 Department of Plant Pathology, University of Georgia, Athens, GA, USA

**Keywords:** halophos, HiVir, ice nucleation, pangenomics, *Pantoea allii*, tailocin, type VI secretion system

## Abstract

*Pantoea allii*, one of four *Pantoea* species known to cause onion centre rot, is infrequently isolated from onion compared to its closely related onion-pathogenic species in the same genus. To better understand the genomic diversity and genetic determinants of pathogenicity in this species, we analysed a collection of 38 *P*. *allii* strains isolated from 2 primary ecological niches, plants and rainwater, across North and South American and African continents using comparative genomics and phylogenetic approaches. Core-genome phylogeny, average nucleotide identity and gene presence–absence analyses revealed three genetically distinct lineages. All strains harboured conserved biosynthetic gene clusters (BGCs) for quorum sensing, carotenoid production, siderophores and thiopeptides. In contrast, two phosphonate BGCs, key determinants of onion pathogenicity, exhibited lineage-specific distributions. Onion-associated strains from lineages 1 and 2 carried the Halophos BGC responsible for onion tissue necrosis and also encoded the *alt* gene cluster that confers tolerance to thiosulfinates. Lineage 3 strains, isolated from both onion and rainwater, either lacked a phosphonate BGC entirely or possessed the HiVir phosphonate BGC. In addition, lineage 3 strains lacked the *alt* cluster altogether. The localization of these virulence genes in the genome varied, with Halophos integrated in the chromosome, HiVir encoded on the conserved Large *Pantoea* Plasmid, and *alt* located on small, variable plasmids (plasmid B). The type IV secretion system (T4SS) and type VI secretion system (T6SS) showed variable genomic architectures, with plasmid-borne T4SSs and two chromosomal T6SS loci differing in conservation and gene content. Additionally, conserved Pantailocin phage islands were detected in most genomes. Overall, this study reveals that while core metabolic and competitive traits are conserved across *P. allii*, virulence-associated loci display lineage-specific distribution, reflecting ecological differentiation and evolutionary plasticity within the species.

**Impact Statement**

This study presents a comprehensive comparative genomic analysis of available genomes of *Pantoea allii*, a known onion pathogen. By examining 38 genomes from strains isolated from plant and water sources across 3 continents, we identified lineage-specific distributions of key virulence genes and conserved genetic traits associated with microbial competition and environmental persistence. These findings reveal the genetic basis of *P. allii* pathogenesis and ecological success, offering broader insights into how plant-associated bacteria persist and compete across diverse habitats.

## Data Summary

Accession numbers for all 38 genomes analysed in this study are listed in Table 1. Complete and draft genome assemblies generated in this study have been submitted to NCBI under BioProject PRJNA1261637.

Figshare DOI: 10.6084/m9.figshare.30866138[1].

**Table 1.** A list of strains used in this study

Strain name	Genome assembly no.	GenBank accession no.	Isolation source	Country of origin	Region	Year isolated	Genome sequence
21GA0466	ASM5056522v1	GCA_050565225.1	Onion	USA	GA	2021	This study
21GA0550	ASM5056618v1	GCA_050566185.1	Onion	USA	GA	2021	This study
21GA0582	ASM5056616v1	GCA_050566165.1	Onion	USA	GA	2021	This study
BD 386	ASM1929520v1	GCF_019295205.1	Onion	ZAF	GP	2003	Public
BD 387	ASM1929516v1	GCF_019295165.1	Onion	ZAF	GP	2003	Public
BD 388	ASM1929517v1	GCF_019295175.1	Onion	ZAF	GP	2003	Public
DOAB1050	ASM1320157v1	GCF_013201575.1, GCA_050857665.1	Wheat	CAN	AB	2017	Public (GCF_013201575.1), this study (GCA_050857665.1)
LMG 24202 (DOAB1035)	ASM5085689v1	GCA_050856895.1	Onion	USA	GA	2002	This study
LMG 24203 (DOAB1036, BD377)	ASM5056520v1	GCA_050565205.1	Onion	ZAF	GP	2004	This study
LMG 24248^T^ (BD390)	ASM230747v1	GCF_002307475.1, GCA_050856905.1	Onion seed	ZAF	GP	2004	Public (GCF_002307475.1), this study (GCA_050856905.1)
BD 381	ASM1929522v1	GCF_019295225.1	Onion	ZAF	GP	2003	Public
BD 382	ASM1929514v1	GCF_019295145.1	Onion	ZAF	GP	2003	Public
BD 383	ASM1929512v1	GCF_019295125.1	Onion	ZAF	GP	2003	Public
BD 389	ASM1929508v1	GCF_019295085.1	Onion	ZAF	GP	2003	Public
MAI 6004	ASM5056516v1	GCA_050565165.1	Onion	URY	CA	2018	This study
MAI 6006	ASM5056612v1	GCA_050566125.1	Onion	URY	CA	2018	This study
MAI 6022	ASM5085821v1	GCA_050858215.1	Onion	URY	CA	2018	This study
OXWO6B1	ASM164113v1	GCF_001641135.1	Oat seed	CAN	SK	2008	Public
19UT001	ASM5056517v1	GCA_050565175.1	Onion	USA	UT	2019	This study
20T×0020	ASM2258540v1	GCF_022585405.1	Onion	USA	TX	2020	Public
BAV3048	ASM3005726v1	GCF_030057265.1	Rain	USA	VA	2013	This study
BAV3051	ASM5056514v1	GCA_050565145.1	Rain	USA	VA	2013	This study
BAV3052	ASM5085628v1	GCA_050856285.1	Rain	USA	VA	2013	This study
BAV3055	ASM3005725v1	GCF_030057255.1	Rain	USA	VA	2013	Public
BAV3060	ASM5056512v1	GCA_050565125.1	Rain	USA	VA	2013	This study
BAV3063	ASM5056510v1	GCA_050565105.1	Rain	USA	VA	2013	This study
BAV3069	ASM5056508v1	GCA_050565085.1	Rain	USA	VA	2013	This study
BAV3070	ASM5056506v1	GCA_050565065.1	Rain	USA	VA	2013	This study
BAV3073	ASM5056504v1	GCA_050565045.1	Rain	USA	VA	2013	This study
BAV3074	ASM5056502v1	GCA_050565025.1	Rain	USA	VA	2013	This study
BAV3077	ASM3005873v1	GCF_030058735.1	Rain	USA	VA	2013	Public
DOAB1051	ASM5085763v1	GCA_050857635.1	Wheat	CAN	AB	2017	This study
Eh250 (CUCPB2048)	Eh250	GCF_046529425.1	Pear shoot	USA	NY	1981	Public
J12b	ASM5056498v1	GCA_050564985.1	Onion	USA	NY	2011	This study
MAI 6007	ASM5085822v1	GCA_050858225.1	Onion	URY	CA	2018	This study
O64c	O64c	GCF_046529495.1	Onion	USA	NY	2011	Public
PNA 200–10	ASM314893v1	GCF_003148935.1	Onion	USA	GA	2000	Public
SJZ147 (strain not available)	ASM782860v1	GCF_007828605.1	Beachgrass rhizosphere	USA	NY	2015	Public

CAN, Canada; URY, Uruguay; USA, United States of America; ZAF, South Africa.

## Introduction

The genus *Pantoea* (order *Enterobacterales*) comprises a group of ubiquitous bacteria capable of colonizing a broad range of ecological niches, including plants, insects, soil and aquatic environments [2,3]. Members of this genus are well known for their diverse interactions with plants, which range from mutualistic relationships as plant growth-promoting endophytes and epiphytes to antagonistic interactions as plant pathogens [4,5]. This ecological flexibility enables *Pantoea* species to persist in both natural and agricultural ecosystems, where they contribute to microbial communities and influence plant health.

Among these species, *Pantoea allii* is relatively understudied despite its ecological and agricultural relevance. First described in South Africa from symptomatic onion (*Allium cepa*) tissues and seeds [6], *P. allii* has been implicated in the onion centre rot disease alongside *Pantoea ananatis*, *Pantoea agglomerans* and *Pantoea stewartii* subsp. *indologenes* [7,11]. This disease complex, which causes foliar necrosis and internal bulb decay [7], affects onions during cultivation and post-harvest storage [8] and remains a persistent threat to onion production worldwide. Despite its involvement in disease, *P. allii* is infrequently isolated, and the genomic basis of its pathogenesis and ecological flexibility remains poorly characterized.

Several virulence-associated loci have been described in onion-pathogenic *Pantoea* species. These include the phosphonate biosynthetic clusters, namely, HiVir [12,14] and Halophos [15], which encode phytotoxins responsible for inducing onion tissue necrosis, as well as the *alt* gene cluster [16], which confers tolerance to onion-derived antimicrobial thiosulfinates. While these loci are well documented in *P. ananatis* [12], *P. stewartii* subsp. *indologenes* [15], and *P. agglomerans* [14], their distribution and functional conservation in *P. allii* are currently unclear.

Beyond plant pathogenicity, interbacterial competition plays a central role in the ecological success of many bacterial species including *Pantoea* [17]. The type VI secretion system (T6SS) is a contact-dependent molecular weapon used to inject antibacterial effectors (toxins) into neighbouring cells and has been shown to modulate both microbial competition and symptom development [18,19]. Genomic analyses of *P. ananatis* and *P. agglomerans* have revealed multiple T6SS gene clusters in each species [20,21]. In both species, the T6SS-1 cluster encoded a functional system involved in interbacterial antagonism [18,19], and in the case of *P. ananatis*, it also contributed to virulence in plants [19]. In addition to the T6SS, *Pantoea* species deploy tailocins, phage tail-like bacteriocins that mediate targeted and efficient elimination of closely related competitors [22]. These systems are being investigated for their potential in microbial control strategies, particularly as safer and more environmentally sustainable alternatives to chemical treatments for bacterial pathogens in agriculture. While well-characterized in related species, the distribution and potential ecological roles of T6SS and tailocins in *P. allii* have not been systematically evaluated.

Ice nucleation activity (INA) is a biological trait by which certain bacteria initiate ice formation in supercooled water at subfreezing temperatures [23]. This phenotype has been documented in *Pantoea* species since the 1980s [24,25]. INA is mediated by the *ina* gene, which encodes a repetitive outer membrane protein that facilitates the spatial organization of water molecules into an ice-like structure on the cell surface [26,27]. INA by plant pathogenic bacteria has been associated with frost injury in plants, which may enhance host susceptibility to colonization by the bacteria [28]. In addition, INA has also been implicated in atmospheric processes, including cloud glaciation and precipitation dynamics [29]. While INA has not been confirmed in *P. allii*, its isolation from rainwater suggests the potential for similar environmental adaptations that may support its persistence and dissemination.

In this study, we performed a comparative genomic analysis of 38 *P*. *allii* strains isolated primarily from plant hosts and rainwater. Accordingly, our objectives were to (i) investigate the phylogenomic structure of *P. allii*, an infrequently isolated species, (ii) characterize the distribution and diversity of virulence- and competition-associated loci and (iii) assess phenotypic traits related to onion pathogenicity and ice nucleation. This work advances our understanding of *P. allii* as both a plant-associated pathogen and an environmentally adapted competitor, and it identifies genetic features that may be leveraged for sustainable disease management.

## Methods

### Culturing

Pure cultures of *P. allii* strains, previously isolated following the methods described in [11,14, 30], were stored in 10% glycerol at −80 °C and were routinely cultured overnight at 28 °C in Lysogeny broth (LB) or LB agar (10 g l^−1^ tryptone, 5 g l^−1^ yeast extract, 10 g l^−1^ sodium chloride or 15 g l^−1^ agar). For ice nucleation assay, strains of *P. allii* were cultured on Reasoner’s 2 agar at 28 °C for 24 h.

### DNA extraction, whole-genome sequencing and genome assembly

Twenty-three *P. allii* strains were sequenced for this study (Table 1). The bacterial cells were pelleted by centrifuging 3 ml overnight culture at 2,500 relative centrifugal force (Eppendorf 5810 R), and genomic DNAs were extracted using Wizard Genomic DNA Purification Kit (Promega). For strains 21GA0466, 21GA0550 and 21GA0582, the Monarch Genomic DNA Purification Kit (New England Biolabs) was used, while Power Water DNA isolation kit (Qiagen) was used for BAV3070.

For strain BAV3070, Duke University Sequencing and Genomic Technologies (Durham, NC) performed whole-genome library preparation and sequencing using Illumina HiSeq4000. Reads with an average per-base quality score <30 and read length <150 bp were filtered out, and the filtered reads were assembled using SPAdes (v3.1.0).

For the remaining strains, library preparation and sequencing were performed by SeqCenter (Pittsburgh, USA) using the Illumina DNA Prep kit with IDT 10 bp UDI indices. The sequencing was carried out on an Illumina NextSeq 2000, generating 2×150 bp paired-end reads. The raw reads were quality-filtered and adapter-trimmed using Trimmomatic (v0.39) using ILLUMINACLIP:NextraPE-PE.fa:2 : 3 : 10 LEADING:3 TRAILING:3 SLIDINGWINDOW:4 : 15 MINLEN:36 as options [31]. *De novo* assembly of the processed reads was performed with SPAdes (v3.15.4) [32]. The quality and completeness of all genome assemblies were evaluated using QUAST (v5.0.2) [33] and BUSCO (v5.2.2) [34], respectively.

Some genomes were closed by performing hybrid assembly of Illumina reads complemented with long reads. Long-read sequencing was performed using a MinION Mk1B device (Oxford Nanopore Technologies) equipped with an R9 Spot-ON flow cell. Library preparation was carried out with the Rapid Barcoding Sequencing Kit (SQK-RBK004), and the flow cell was primed using the EXP-FLP002 Flow Cell Priming Kit, following the manufacturer’s protocols. Basecalling of raw signal data was conducted using Guppy (Oxford Nanopore Technologies), and demultiplexing of barcoded reads was performed with qcat (https://github.com/nanoporetech/qcat).

Nanopore reads were initially trimmed and filtered for quality using FiltLong (https://github.com/rrwick/Filtlong) with options --min_length 1000 --keep_percent 95. A subset of high-quality reads was randomly sampled to a defined coverage depth to standardize input for downstream assembly. Multiple draft assemblies were generated using distinct long-read assemblers, including Flye [35], MiniPolish [36] and Raven [37]. These assemblies were then reconciled into a single consensus genome using Trycycler [38], which integrates structural variation across assemblies to produce a refined consensus. The resulting genome was polished in two stages: first with Medaka (https://github.com/nanoporetech/medaka) using the Nanopore reads, followed by additional polishing with NextPolish [39] using Illumina short reads that had been quality-filtered with FASTP [40]. Final curation steps were performed with custom scripts, which included removal of contaminant sequences (e.g. phiX174), renaming of contigs based on descending sequence length and reorientation of sequences to place key replication genes, such as *dnaA*, near the beginning of the positive strand.

Genome quality was assessed through annotation with the NCBI Prokaryotic Genome Annotation Pipeline (PGAP) [41] and evaluation of proteome completeness using BUSCO [34]. To validate assembly accuracy, an independent *de novo* assembly was also generated using Unicycler [42] and compared with the consensus assembly using ‘dnadiff’ from the MUMmer package.

### Pangenome analysis and construction of recombination-free core-genome phylogenetic tree

In addition to 23 newly assembled genomes of this study, 15 additional *P. allii* genomes were downloaded from the NCBI for pangenome analysis. The strain name, metadata and the GenBank accession numbers of genomes used in the analysis are listed in Table 1. The total of 38 genomes was annotated using PROKKA (v1.14.5) [43], and the resulting annotation files (gff) were analysed for the pangenome of *P. allii* using Roary (v3.13.0) [44]. Roary was run with options -e --mafft to generate an alignment of core genes (present in >95% of genomes) which was used to construct core-genome phylogenetic tree using FastTree v2.1.11 [45]. To build a recombination-free phylogenetic tree, recombination regions of core genes were identified using ClonalframeML (v1.12) with default parameters [46], and the regions were masked by running cfml-maskrc script (https://github.com/kwongj/cfml-maskrc) prior to reconstructing a recombination-free tree. The recombination-free core-genome phylogenetic tree was constructed using FastTree.

### Average nucleotide identity, digital DNA–DNA hybridization analysis and Life Identification Number assignment

Nucleotide sequences of 38 *P*. *allii* genome assemblies were analysed for their genome distance to each other. Pairwise average nucleotide identity (ANI) was calculated using FastANI (v1.33) [47] with default settings, and resulting ANI matrix was visualized as a heatmap using an R script available at https://github.com/spencer411/FastANI_heatmap. Digital DNA–DNA hybridization (dDDH) between the type strain *P. allii* LMG24248^T^ (GCF_002307475.1) and 38 genomes of this study was conducted using Genome-to-Genome Distance Calculator v3.0 through Type Strain Genome Server (TYGS) (available at ggdc.dsmz.de/ggdc.php#). The dDDH values calculated from Formula 2 [sum of all identities found in high-scoring segment pairs (HSPs) divided by overall HSP length] were selected. Life Identification Numbers (LINs) were assigned to each genome through the genomeRxiv webserver (http://www.genomerxiv.org), a more comprehensive and updated version of the legacy LINbase web server [48].

### Identification of secondary metabolite biosynthetic gene clusters and ice nucleation gene

Secondary metabolite biosynthetic gene clusters (BGCs) in the genomes of *P. allii* were determined using antiSMASH included in the Beav (v1.4.0) pipeline [49]. Previously characterized HiVir and Halophos BGCs responsible for producing onion necrosis factors in *P. ananatis* [12] and *P. stewartii* subsp. *indologenes* [15] and ice nucleation protein encoding gene of *P. ananatis* PNA 97-1 [50] were searched against 38 *P*. *allii* genomes using nucleotide blast+ (v2.14.1) search [51]. Presence and absence data of virulence gene clusters were included in the metadata table (Table S1, available on figshare).

### Identification of known type secretion systems

The presence of type III secretion system (T3SS), type IV secretion system (T4SS) and T6SS was identified using Beav v1.4.0 pipeline [49]. A type secretion system was deemed present when a full quorum of core or mandatory genes of a system was met. For draft genomes, a quorum of mandatory genes was relaxed by one. Conjugative elements were further screened from Prokka protein annotations of 38 *P*. *allii* genomes by MacSyFinder [52] using CONJScan models [53].

### Detection of phage and Pantailocin islands

Genome-wide detection of phage genes and islands was performed on the *P. allii* genomes using PHASTEST (https://phastest.ca/). The Pantailocin island, previously characterized in *P. ananatis* ATCC 35400 and *P. stewartii* subsp. *indologenes* ICMP 10132 [22], is flanked by the *rpoD* and *sulP* genes. This region was extracted from the nine closed *P. allii* and compared to those of *P. ananatis* and *P. stewartii* by clinker analysis described below.

### Gene cluster homology and synteny analysis

T6SS loci and Pantailocin gene clusters from nine closed-genome *P. allii* strains were extracted in GenBank format for comparative analysis. Type VI loci were compared to those of *P. ananatis* DZ-12 (GCA_003849975.1) and *P. agglomerans* pv. *betae* strain 4188 (GCF_001662025.2), while Pantailocin gene clusters were compared to those of *P. ananatis* ATCC 35400 (GCF_029433965.1) and *P. stewartii* subsp. *indologenes* ICMP 10132 (GCF_029433915.1), using Clinker (https://cagecat.bioinformatics.nl/tools/clinker) with default settings [54]. Collinearity and homology of plasmid Bs from closed genome *P. allii* strains were compared using MAUVE alignment tool [55].

### Replicon comparison by BRIG

The genome similarity of nine closed genome strains was compared by replicons using BRIG (v0.95) [56]. *P. allii* LMG24248^T^ was used as a reference sequence (central ring) for chromosomes, whereas Eh250 was used as a central ring for Large *Pantoea* Plasmid (pLPP) and plasmid B. The genomic location of HiVir, Halophos and *alt* (thiosulfinate tolerance) gene clusters was manually searched and indicated on the ring diagrams.

### Red onion scale necrosis assay

Single bacterial colonies were inoculated into 3 ml of LB broth and incubated overnight at 28 °C with shaking at 200 r.p.m. To prepare the bacterial inoculum for the red onion scale necrosis (RSN) assay, the overnight cultures were pelleted and resuspended in sterile 1× PBS to an OD of 0.3 at 600 nm. The RSN assay was performed according to the previously described protocol [57]. The onion scales were visually assessed and imaged at 4 days post-inoculation (dpi). Two independent RSN assays were conducted.

### Ice nucleation assay

INA was assessed using a droplet freezing assay adapted from [58]. A bacterial suspension with an OD_600_ of 0.2 (~10^8^ c.f.u. ml^−1^) was prepared in molecular grade water. The suspensions were incubated at 4 °C for at least 1 h. Thirty 20 µl droplets were then placed onto a parafilm boat floating on a glycerol bath maintained at −2 °C in a cooling thermostat (Lauda Alpha RA24, LAUDA-Brinkmann, Delran, NJ, USA). The droplets were cooled from −2 to −12 °C at a rate of −1 °C every 10 min. For each strain, the experiment was performed in triplicate and repeated three independent times, and the average freezing-onset temperature was recorded.

## Results

### *P. allii* forms three genetically distinct lineages with high genomic similarity

Genetic diversity and phylogenetic relationships among environmental and plant-associated *P. allii* strains were investigated by analysing 15 publicly available and 23 newly sequenced genomes. Six genomes of strains, LMG24248 (lineage 1, onion, South Africa), DOAB1035 (lineage 1, onion, USA), MAI6022 (lineage 2, onion, Uruguay), DOAB1050 (outgroup close to lineage 3, wheat, Canada), BAV3052 (lineage 3, rainwater, USA), DOAB1051 (lineage 3, wheat, Canada) and MAI6007 (lineage 3, onion, Uruguay), were closed using hybrid assemblies in this study, while two additional closed genomes were obtained from public databases (Table 1). These six strains were selected to represent the diversity of the dataset, with consideration given to lineage assignment, source of isolation (plant vs. environment) and geographic origin. BUSCO analysis indicated 100% completeness of the newly sequenced genomes (Table S2). The pangenome of the 38 *P*. *allii* strains comprised a total of 10,127 orthologous and 2,950 unique genes. Of the total orthologous genes, 3,700 were core genes (present in 99–100% of genomes), 87 were soft-core genes (present in 95–99% of genomes), 1,400 were shell genes (present in 15–95% of genomes) and 4,940 were cloud genes (present in 0–15% of genomes) (Fig. S1A). The average number of core genes per *P. allii* genome in this study was 3,787, while the total gene count averaged 4,591. The 38 *P*. *allii* strains shared ANI ranging from 98% to 100 % (Fig. S1B) and dDDH values ranging from 89% to 100% (Table S3) . The genome size varied from 4.7 to 5.2 Mb with an average GC content of 53% (Table S4).

**Fig. 1.**
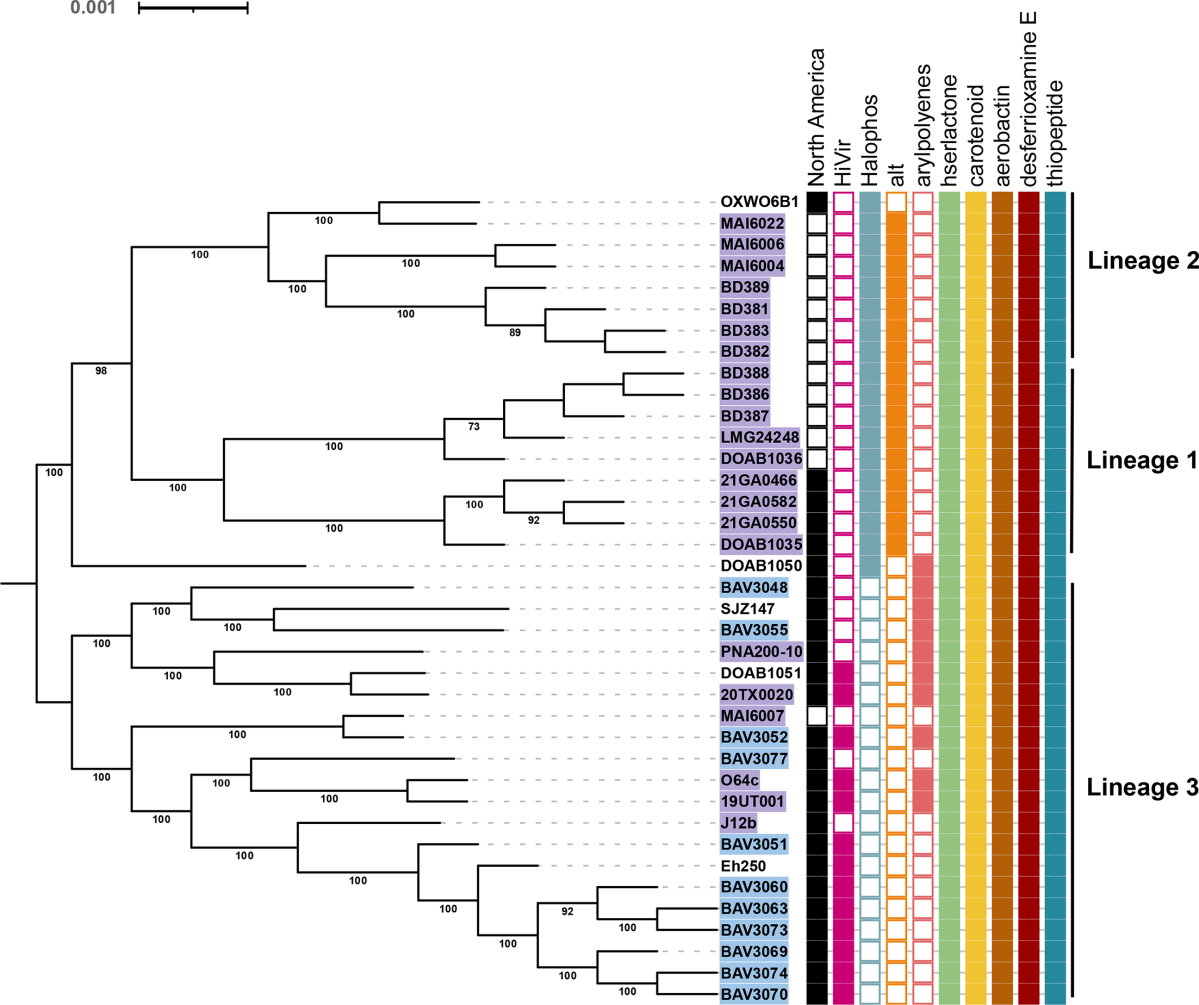
Three distinct lineages are present in *P. allii*. A recombination-free, approximately maximum-likelihood phylogenetic tree constructed based on 3,700 core genes shared among 38 *P. allii* strains using FastTree (v2.1.11) and ClonalFrameML (v1.12). The general time reversible nucleotide substitution model was selected as the best-fit evolutionary model, and the robustness of groupings was assessed by the approximate likelihood-ratio test. The tree was midpoint-rooted and edited in iTOL (v6.8, available at https://itol.embl.de/). Strain names are highlighted in blue (rainwater) and purple (onion-associated) to indicate their isolation source. The coloured boxes next to strain names indicated the geographic origin (North America) and the presence of virulence genes (HiVir, Halophos), antimicrobial resistance genes (*alt*, *al*licin *t*olerance) and BGCs for pigment (aryl polyenes, carotenoid), quorum sensing (acylated homoserine lactone), siderophores (aerobactin, desferrioxamine E) and antimicrobials (thiopeptide).

A phylogeny based on the core-genome (*n*=3,700) largely divided *P. allii* strains into three lineages with a singleton represented by strain DOAB1050, which is basal to the node representing the most recent common ancestor of lineages 1 and 2 (Fig. 1). Lineages 1 and 2 each have a relatively limited sub-clade structure with only three to four sub-clades with high bootstrap support. Lineage 3 instead has 18 highly supported subclades. The sub-clades of each lineage were supported by groupings based on ANI (Fig. S1B) and gene presence–absence profiles (Fig. S2, Table S12). Strains belonging to lineages 1 and 2 were isolated from various countries including South Africa, Uruguay and the USA (Georgia), whereas lineage 3 was comprised of strains predominantly isolated from the USA, with the exceptions of one strain isolated from Uruguay (MAI6007) and the other from Canada (DOAB1051). The three main lineages were circumscribed in the genomeRxiv web server [48] as follows: lineage 1 (76_A_6_B_13_C_0_D_0_E_0_F_0_G_0_H_0_I_0_J_0_K_), lineage 2 (76_A_6_B_13_C_0_D_0_E_0_F_0_G_0_H_0_I_0_J_1/11_K_) and lineage 3 (76_A_6_B_1_C_0_D_1_E_0_F_0_G_0_H_0_I_0_J_2/3/5/6/7/8/9/10/12/13_K_), whereby the high number of LINs at position K is due to the high number of sub-clades belonging to lineage 3 (Table S5).

### Presence of broadly conserved secondary BGCs

Analysis of 38 *P*. *allii* genomes using antiSMASH [49] identified secondary metabolite BGCs with more than 70% protein sequence homology to known homoserine lactone, aryl polyene, carotenoid, aerobactin, desferrioxamine E and thiopeptide clusters (Table S6). With the exception of aryl polyene, all identified BGCs were conserved across the three *P. allii* lineages (Fig. 1). The homoserine lactone BGC in *P. allii* contained the quorum sensing genes *esaI* acyl-homoserine lactone (AHL) synthase and *esaR* transcription factor which mediate the synthesis of and regulation by AHL quorum sensing molecules [59]. Carotenoid BGCs which encode lipid-soluble pigments involved in photoprotection and oxidative stress resistance were universally identified on the Large *Pantoea* Plasmid [60,61]. These clusters included a conserved *crt* operon (*crtE*, *crtX*, *crtY*, *crtI*, *crtB* and *crtZ*). In contrast, aryl polyene BGCs, which encode structurally similar pigmented metabolites but are synthesized via type II polyketide synthases [62], were only detected in a subset of *P. allii* strains belonging to lineage 3. Additionally, two siderophore BGCs resembling those of aerobactin and desferrioxamine E were identified [63], suggesting the presence of iron scavenging mechanism in *P. allii*. A BGC encoding a ribosomally synthesized and post-translationally modified thiopeptide was also detected [64], indicating the potential for antimicrobial compound production in *P. allii*.

### Lineage-specific distribution of phosphonate biosynthetic and thiosulfinate tolerance (*alt*) gene clusters

To date, two phosphonate BGCs have been functionally characterized for their role in the pathogenicity of *Pantoea* species in onion. These BGCs, named Halophos [15] and HiVir [12], encode phytotoxic phosphonate compounds that induces necrosis in onion tissue. Halophos was exclusively identified in *P. allii* strains from lineages 1 and 2, which are predominantly onion-associated, with the exceptions of strains DOAB1050 and OXWO6B1, isolated from wheat and oat seed, respectively (Table 1). The Halophos-positive strains were collected from North and South America and Africa. In contrast, HiVir was found exclusively in lineage 3 strains, isolated from rainwater, onion, pear shoot and wheat. Notably, seven strains from lineage 3 (BAV3048, BAV3055, BAV3077, MAI6007, PNA200-10, J12b and SJZ147) lacked both phosphonate BGCs, and no strain was found to contain both the Halophos and HiVir gene clusters simultaneously. However, strain MAI6022 harboured a Halophos and an additional phosphonate BGC designated ‘*pgb*’ which is distinct from Halophos and HiVir. The *pgb* was previously identified [13] and shown not to impact the onion necrosis phenotype in *P. ananatis* [65].

The *alt* tolerance gene cluster which encodes enzymes that alleviate thiosulfinate-associated thiol stress resulting from onion necrosis [16] was present only in onion-associated, Halophos-positive strains from lineages 1 and 2. This gene cluster was absent in DOAB1050, a strain isolated from wheat and OXWO6B1, a potato late blight biocontrol strain which inhibits the growth of *Phytophthora infestans* [66].

### Onion pathogenicity assays show that necrosis is more frequently observed among strains harbouring a phosphonate BGC

**Fig. 2.**
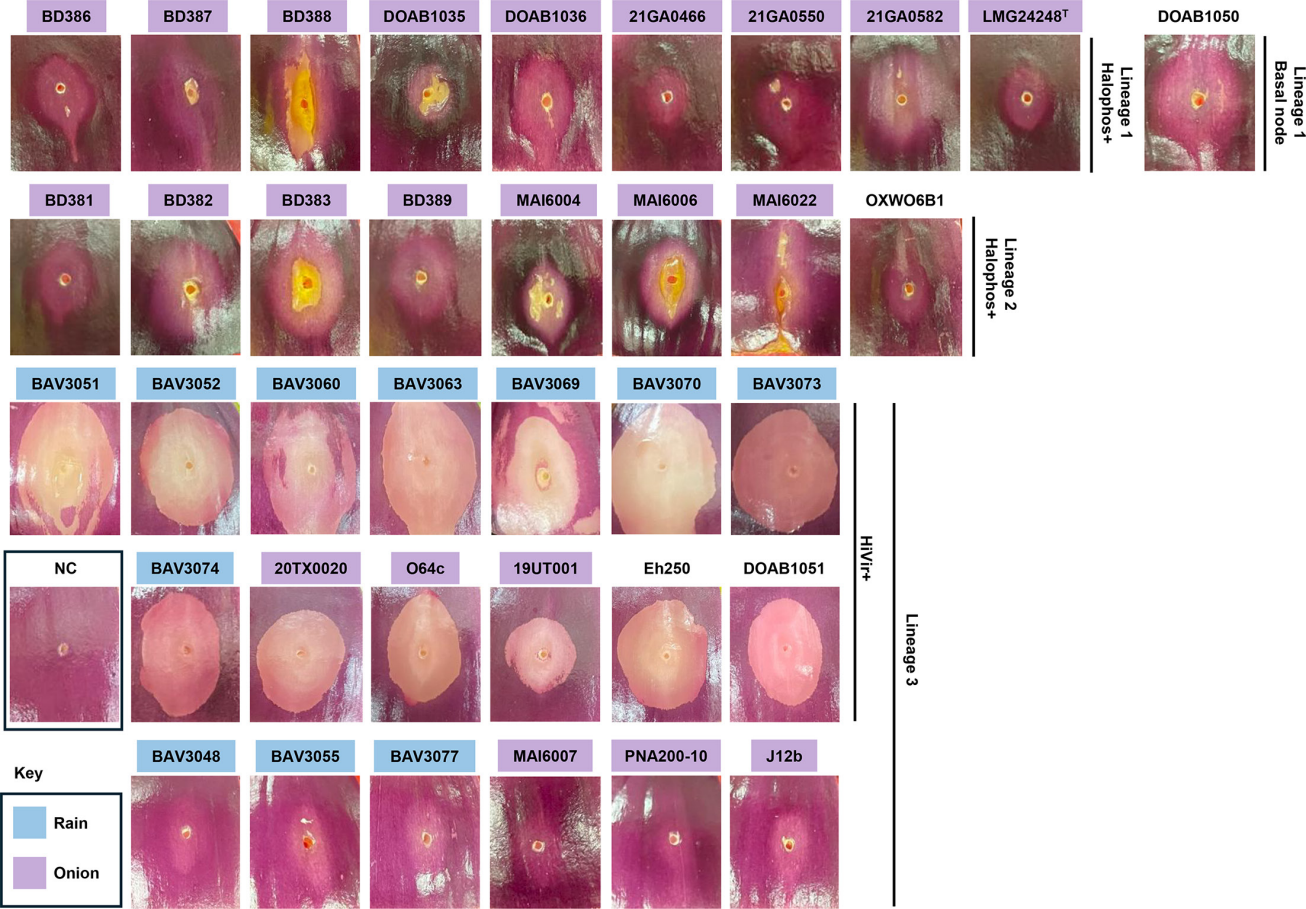
Presence of HiVir and Halophos phosphonate BGCs is associated with distinct RNS phenotypes. Pathogenicity of 37 *P. allii* strains was assessed using detached red onion scales. Four dpi, HiVir-positive strains, isolated from rainwater and onion tissues and belonging to lineage 3, caused white clearing (necrotic) zones. In contrast, some Halophos-positive strains, exclusively from lineages 1 and 2, produced a yellow, sunken necrosis phenotype. Strains lacking both HiVir and Halophos BGCs did not induce necrosis, although faint lightening of the red onion scales was occasionally observed. No symptoms were observed on scales inoculated with the negative control (NC). Strain names are coloured blue (rainwater) and purple (onion-associated) to indicate their source of isolation.

To assess the virulence of *P. allii* strains, the pathogenicity of 37 strains was tested using detached fleshy red onion scales; one strain was not available (Fig. 2). Four dpi, distinct necrosis patterns were observed. HiVir-positive *P. allii* strains caused a characteristic oval-shaped white clearing zone on the onion scales, while some Halophos-positive strains, such as BD388, MAI6004 and MAI6022, produced a sunken yellow necrotic lesion (Fig. 2). In addition to these distinct necrotic patterns, faint discolouration was observed around the inoculation point for certain Halophos-positive strains as well as for strains that lacked both HiVir and Halophos BGCs. No symptoms or discolouration was observed on the red onion scales inoculated with 1× PBS buffer (Fig. 2).

### Halophos, HiVir and *alt*-specific genomic localization on chromosomes and plasmids

**Fig. 3.**
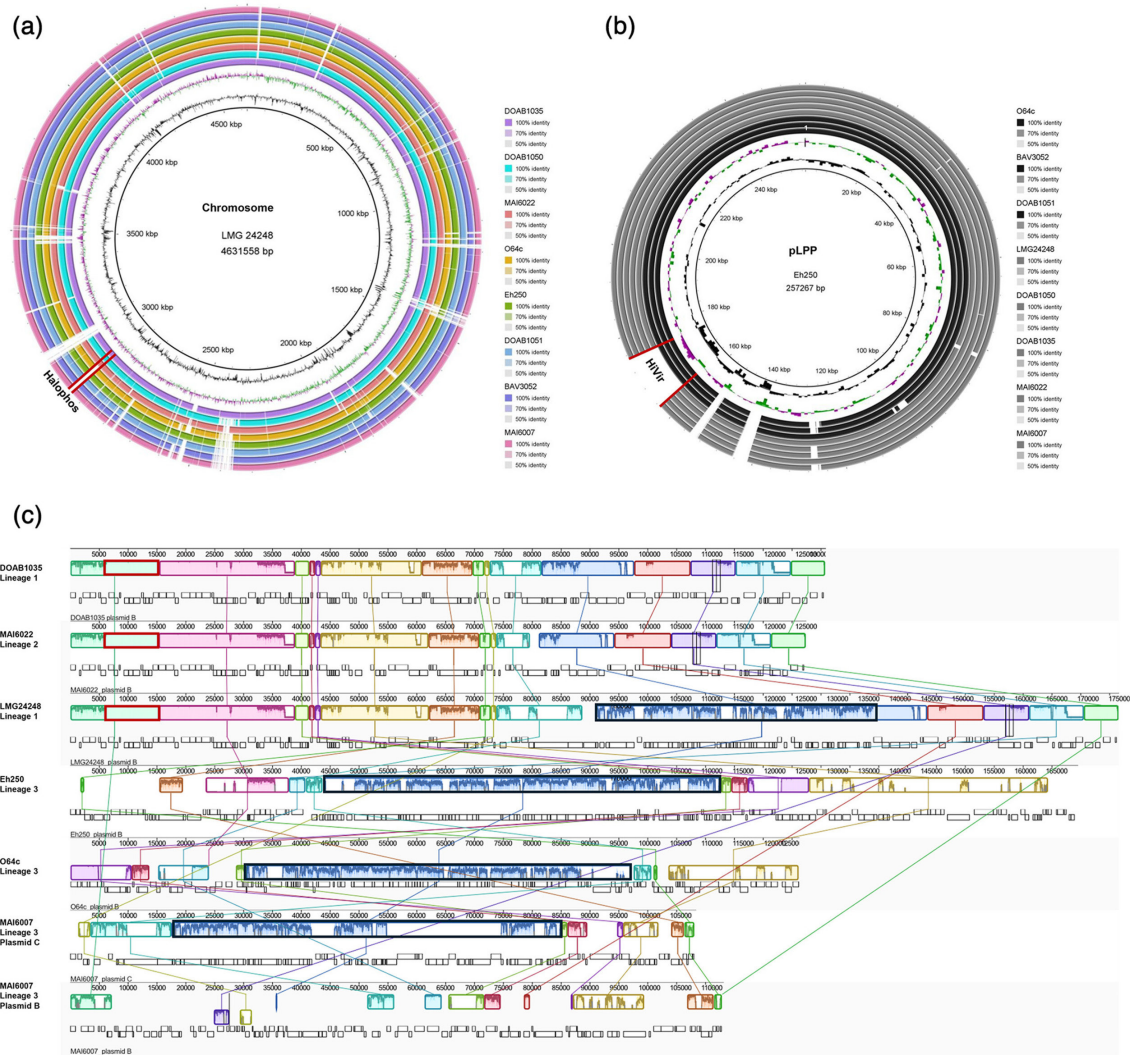
Genomic localization of Halophos, HiVir and *alt* gene clusters on chromosomes and plasmids. The genomic locations of onion virulence gene clusters (HiVir, Halophos) and the antimicrobial resistance gene cluster (*alt*) were investigated in nine closed-genome *P. allii* strains representing each lineage, using BRIG [56] and MAUVE [55] analyses. The analyses revealed that (**a**) Halophos is located on the chromosome, (**b**) HiVir on the large *Pantoea* plasmid (pLPP) and (**c**) the *al*licin *t*olerance cluster (*alt*) is carried on plasmid B. A high degree of conservation was observed for the chromosome and pLPP1 across strains, while plasmid B was more variable. In lineages 1 and 2, plasmid B carried the *alt* cluster (red box, mint coloured block) and showed similar sequence content, whereas plasmid B in lineage 3 strains lacked *alt* and was more conserved within the lineage but divergent from those of lineages 1 and 2. Strain MAI6007 carried an extra replicon, plasmid C shared conjugate T4SS genes (black box, dark blue block) on plasmid Bs of LMG24248^T^, Eh250 and O64c.

To investigate the genomic organization of key onion pathogenicity gene clusters, the location of Halophos, HiVir and *alt* was compared across nine *P. allii* strains with closed genomes. The Halophos gene cluster was located on the chromosome (Fig. 3a), whereas the HiVir cluster was found on the Large *Pantoea* Plasmid which was identified by the presence of carotenoid biosynthesis genes (Fig. 3b). The *alt* gene cluster, associated with thiosulfinate stress tolerance, was present on plasmid B in Halophos-positive strains (DOAB1035, LMG24248 and MAI6022) but absent from plasmid B in HiVir-positive strains (Eh250, MAI6007 and O64c). Plasmid B varied in size, ranging from 113.1 to 174.5 kb, and exhibited substantial differences in gene content between Halophos-positive and HiVir-positive strains (Fig. 3c). Among the nine strains with closed genomes, four (DOAB1035, Eh250, LMG24248 and O64c) contained three replicons: a chromosome, the Large *Pantoea* Plasmid (LPP) and plasmid B (pB). Three strains (BAV3052, DOAB1050 and DOAB1051) carried only two replicons, the chromosome and the Large *Pantoea* Plasmid. In contrast, strains MAI6007 and MAI6022 carried an additional replicon, plasmid C, measuring 108.3 and 66.8 kb, respectively. Plasmid C from MAI6007 included distinct genes, along with plasmid replication genes and remnants of plasmid B-associated sequences (Fig. 3c), while plasmid C from MAI6022 did not show this overlap (data not shown).

### Presence of T4SS and T6SS in *P. allii*

Secretion systems often play a crucial role in bacterial interactions with hosts and other microbes, influencing virulence, competition and environmental adaptation. To assess the presence and distribution of secretion systems in *P. allii*, the 38 genomes were analysed using Beav (v1.4.0) [49]. While the Hrp-type T3SS, typically associated with plant pathogenicity, was absent, genes encoding conjugative T4SS and T6SS were identified on plasmids and chromosomes of *P. allii*, respectively (Table S7). To further investigate the gene synteny and architecture of T4SS and T6SS clusters, nine strains with closed genomes were selected for MAUVE [55] and clinker [54] analyses, respectively. Conjugative T4SS genes were primarily localized on plasmid B, represented by the dark blue block in the MAUVE alignment (Fig. 3c). Beav’s TXSS scan (Table S7) and CONJScan by MacSyFinder [52] predictions indicated that plasmid B of strains LMG24248, O64c and Eh250 carried a structurally complete T4SS cluster, whereas plasmid C of MAI6007 also harboured a complete T4SS (Table S8). In contrast, no T4SS genes were detected on plasmid B of DOAB1035 and MAI6022, nor on the LPP of any *P. allii* strain.

**Fig. 4.**
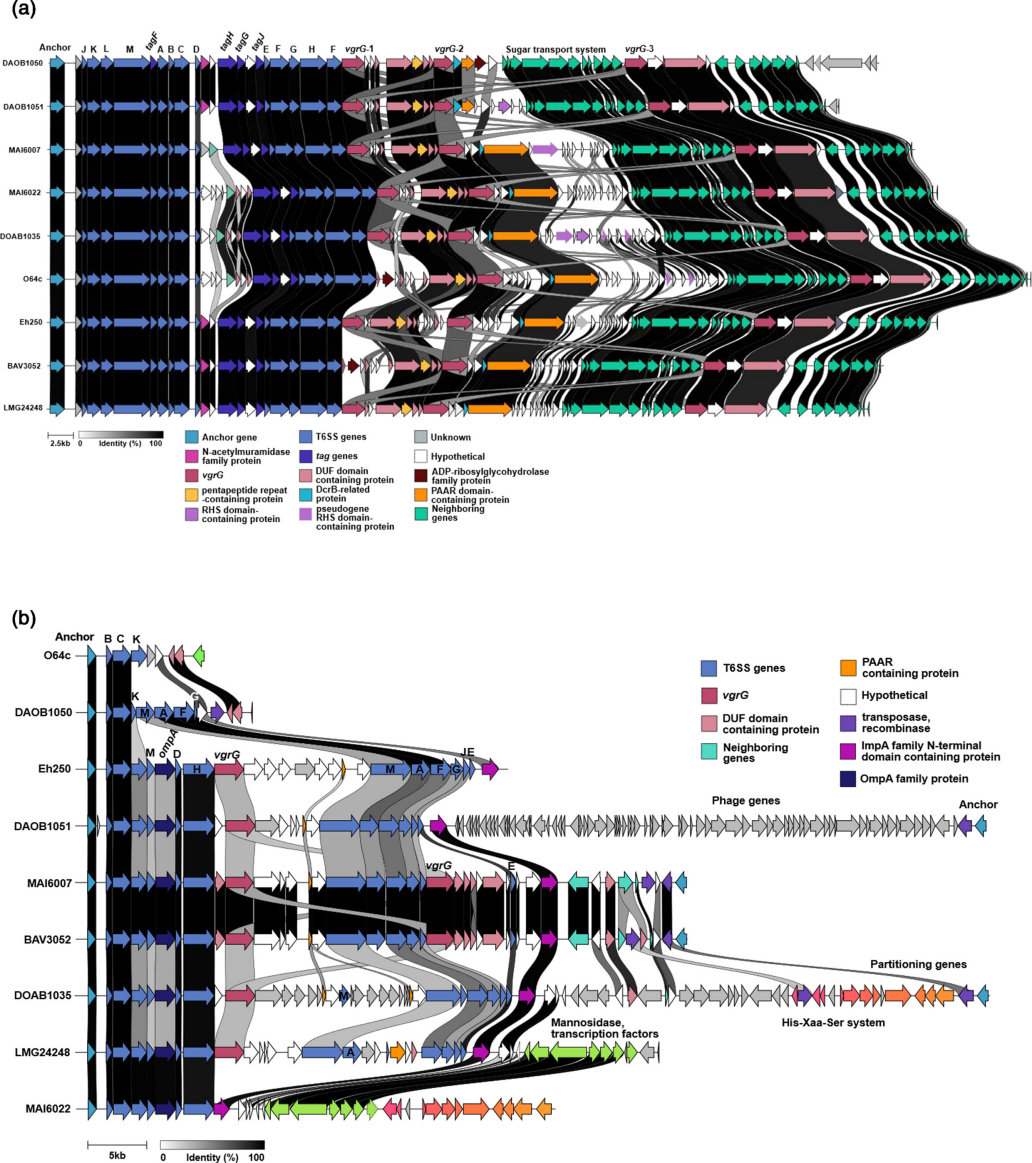
Comparative analysis of two chromosomal T6SS loci in *P. allii* reveals differences in gene content conservation. Two chromosomal T6SS loci were identified in nine closed genome *P. allii* strains and compared using clinker [54] to assess gene content and synteny. Genes are represented as arrows and coloured by predicted function. (**a**) T6SS-I included a complete set of conserved core structural genes [*tssA–M*, *hcp* (*tssD*), *vgrG* (*tssI*) and *clpV* (*tssH*)] arranged in a consistent synteny across all strains, while (**b**) T6SS-II was highly variable in structural gene content among strains. Only MAI6007 and BAV3052 possessed a nearly complete set of core T6SS genes and other strains contained partial clusters with a reduced number of core structural genes.

T6SSs have been found to play a key role in bacterial competition and interactions with plant hosts. In *P. allii*, two chromosomal T6SS loci, T6SS-I and T6SS-II, were identified across the analysed genomes. T6SS-I was positioned downstream of an EmmdR/YeeO family multidrug/toxin efflux MATE protein (LMG24248 CP193910, locus tag: ACRPH8_08530), while T6SS-II was located downstream of a glycerophosphodiester phosphodiesterase family protein (LMG24248 CP193910 locus tag: ACRPH8_06730). These T6SS loci were flanked by tRNA-Asn and tRNA-Arg, respectively. Analysis using Beav’s TXSS scan predicted that T6SS-I contained a structurally complete T6SS gene cluster (Tables S7 and S10, Fig. 4a), while T6SS-II exhibited varying degrees of completeness across strains (Tables S7 and S10, Fig. 4b). Among the nine strains with closed genomes, only MAI6007 and BAV3052 harboured a complete T6SS-II gene cluster (~52 kb). In contrast, O64c (10.1 kb) and DOAB1050 (14.1 kb) contained the shortest T6SS-II loci, while DOAB1051 (76.5 kb) and DOAB1035 (77.8 kb) had the largest. The expansion of T6SS-II in DOAB1051 was attributed to the integration of phage-related genes, whereas in DOAB1035, the presence of genes encoding hypothetical proteins, integrases, transposases and plasmid replication proteins contributed to its increased size (Fig. 4b). Notably, plasmid replication genes were also detected within the T6SS-II loci of MAI6022 and DOAB1035 (Fig. 4b).

Compared to T6SS-II, T6SS-I exhibited greater conservation across *P. allii* strains. Downstream of the structural and T6SS-associated (tag) genes which were absent in T6SS-II, three distinct islands consist of *vgrG* (spike protein encoding gene), and *vgrG*-associated genes were identified, encoding hypothetical and domain of unknown function (DUF)-containing proteins (Fig. 4a, Table S9). However, in strains O64c and BAV3052 (Fig. 4a), the first *vgrG* gene was predicted to be a pseudogene. Despite this, the downstream genes encoding pentapeptide repeat (COG1357), DUF3540 (pfam12059) and DUF4150 (pfam13665) domain-containing proteins remained intact in all nine closed genomes. The second *vgrG* (*vgrG*-2) island contained the highest number of hypothetical protein-encoding genes and was enriched in genes that encode RHS domain- or repeat-containing proteins (Fig. 4a, Table S9). In DOAB1035 and MAI6007, some RHS domain-containing protein encoding genes were predicted to be pseudogenes (Fig. 4a, Table S9). Among the conserved genes on this island, DcrB-related and PAAR (also DUF6531) domain-containing protein encoding genes were consistently present. The PAAR domain-containing proteins also carried an RHS domain or element. In DOAB1050, PAAR domain-containing protein also harboured a domain (pfam03496) found in actin-ADP-ribosylation toxin followed by an ADP-ribosylglycohydrolase family protein. The third *vgrG* island (*vgrG*-3) was located downstream of genes encoding PTS fructose transporter and ABC transporter proteins and was the most highly conserved *vgrG* island of the three, consisting of DUF6531 domain-containing and hypothetical protein encoding genes (Fig. 4a, Table S9).

### Presence of Pantailocin islands in *P. allii*

**Fig. 5.**
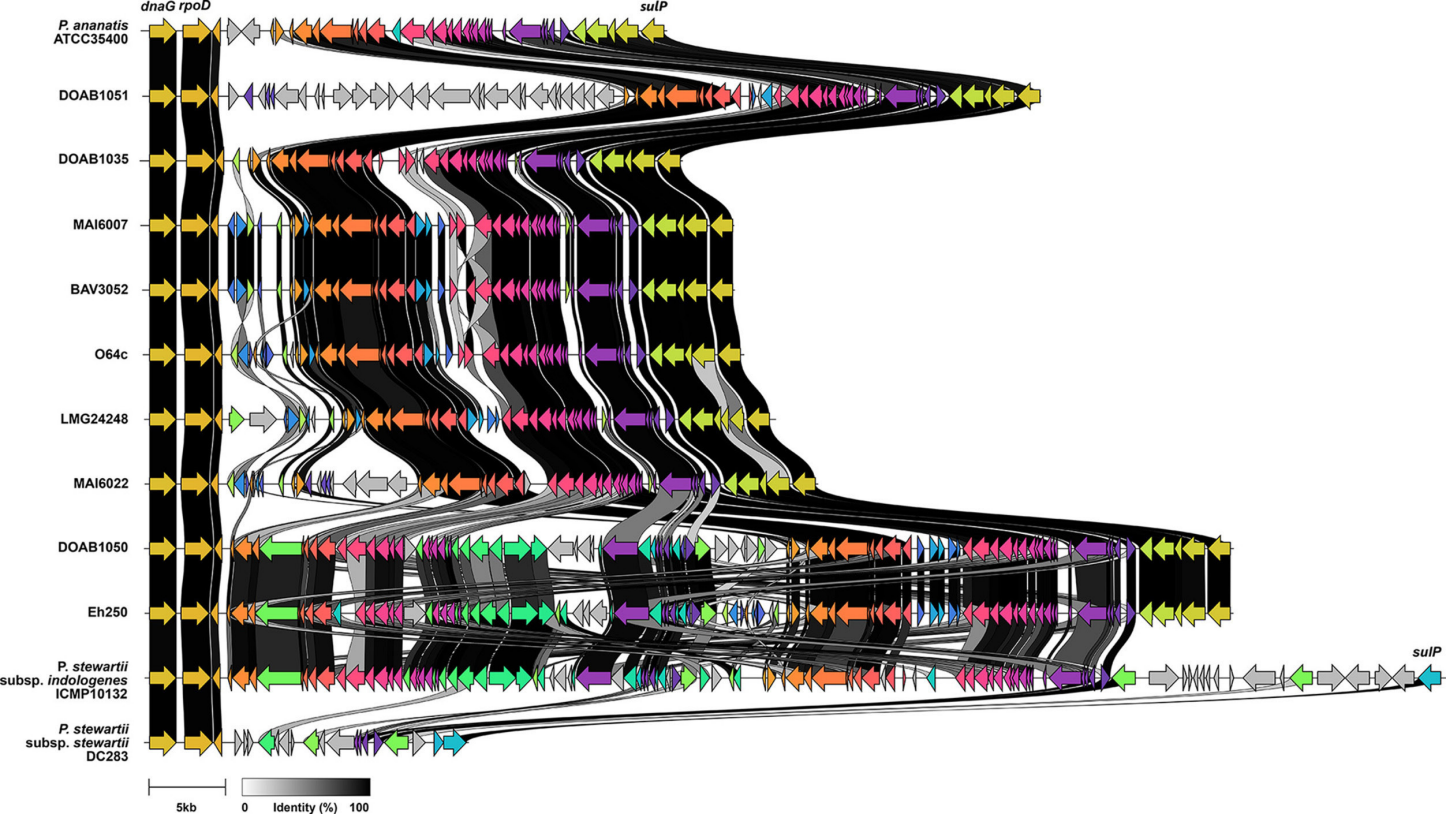
*P. allii* strains harbour the Pantailocin island. The Pantailocin island was initially detected as a prophage island by PHASTEST in *P. allii* strains, located in a conserved chromosomal region flanked by *rpoD* and *sulP* genes. Clinker analysis of the island to the functionally characterized Pantailocin island in *P. ananatis* ATCC 35400 and *P. stewartii* subsp. *indologenes* ICMP10132 revealed high homology. The islands in *P. allii* strains DOAB1035, DOAB1051, BAV3052, O64c, LMG24248, MAI6007 and MAI6022 were most similar to the Pantailocin island found in *P. ananatis*, while those in DOAB1050 and Eh250 were more closely aligned with the island found in *P. stewartii*.

Prophage islands were identified in the genomes of 38 *P*. *allii* strains using PHASTEST [67], which assesses prophage completeness based on phage structural genes (Table S11). Most strains contained at least one intact prophage island. However, no ‘intact’ prophage islands were detected in the draft genomes of BAV3051, 21GA0446, 21GA0550, 21GA0582 and MAI6006. A manual alignment and clinker analyses of intact prophage islands in nine closed genomes revealed a conserved genomic context. These islands ranging in size between 35 and 71 kb were consistently flanked by *rpoD* (LMG24248 CP193910 locus tag: ACRPH8_03485) and *sulP* (LMG24248 CP193910 locus tag: ACRPH8_03230) genes (Fig. 5). Additionally, they shared homology with the Pantailocin island previously identified in *P. ananatis* ATCC 35400 and *P. stewartii* subsp. *indologenes* ICMP10132 [22]. Interestingly, in DOAB1050 and Eh250, the Pantailocin island closely resembled that of *P. stewartii* subsp. *indologenes* ICMP10132, an expanded phage island compared to its counterpart in *P. ananatis* ATCC 35400. In contrast, the Pantailocin islands in BAV3052, DOAB1035, DOAB1051, LMG24248, MAI6007, MAI6022 and O64c were more similar to that of *P. ananatis* ATCC 35400 which exhibited partial homology to the Pantailocin islands in DOAB1050, Eh250 and *P. stewartii* subsp. *indologenes* ICMP10132. Additionally, DOAB1051 Pantailocin island contained extra phage genes (grey arrows) that lacked homology to the rest of phage genes found in *P. allii*, *P. ananatis* ATCC 35400 and *P. stewartii* subsp. *indologenes* ICMP10132 (Fig. 5).

### Widespread INA among *P. allii* strains

Homologues of the ice nucleation protein-encoding *InaZ* gene were detected in all 38 *P*. *allii* genomes, while INA was observed in 24 out of the 26 *P. allii* strains that were available for testing. 80% of these strains exhibited a freezing onset temperature of −10 °C or higher (Table 2). Notably, strain Eh250 displayed the highest activity with a freezing onset temperature of −4.0 °C. Strains BD389 and 20TX0020 did not exhibit INA within the temperature range tested, although the *ina* gene was found in the respective genomes as in all other strains. It is possible that INA expresion in these strains is too weak to be measurable under the conditions used for testing [30].

**Table 2.** Average ice nucleation onset temperature (°C) of *P. allii* strains

Strain	Source	Presence of INA gene	Average INA onset temp (°C)
21GA0466	Onion	Y	−9
21GA0550	Onion	Y	−10.3
21GA0582	Onion	Y	−10
BD386	Onion	Y	−10
BD387	Onion	Y	−10.3
BD388	Onion	Y	−10
DOAB1035	Onion	Y	−10
DOAB1036	Onion	Y	−10
LMG24248	Onion	Y	−10
BD381	Onion	Y	−10
BD382	Onion	Y	−8.3
BD383	Onion	Y	−10.7
BD389	Onion	Y	n/a
DOAB1050	Wheat	Y	−11
MAI6004	Onion	Y	−10
MAI6006	Onion	Y	−10.7
MAI6022	Onion	Y	−10.7
OXW06B1	Oat seed	Y	−11
19UT001	Onion	Y	−9.7
20T×0020	Onion	Y	n/a
BAV3048*	Rain	Y	−12
BAV3051*	Rain	Y	−10
BAV3052*	Rain	Y	n/a
BAV3060*	Rain	Y	−12
BAV3063*	Rain	Y	−10
BAV3069*	Rain	Y	−10
BAV3070*	Rain	Y	−10
BAV3073*	Rain	Y	−10
BAV3074*	Rain	Y	-8
BAV3077*	Rain	Y	−10
DOAB1051	Wheat	Y	−10
Eh250	Pear shoot	Y	-4
J12b	Onion	Y	−7.7
MA16007	Onion	Y	−11.3
O64c	Onion	Y	−10
PNA200-10	Onion	Y	−10
SJZ147	Beachgrass rhizosphere	Y	n/d

Strains marked with an asterisk (*) have their average ice nucleation temperatures reported in Failor *et al*. [30]. n/a indicates that no ice nucleation was observed under the current experimental conditions. n/d indicates that ice nucleation data are unavailable due to the absence of strain.

## Discussion

*Pantoea* species are broadly distributed in diverse environments and are frequently associated with plants as both epiphytes and endophytes [2,4, 5]. Several species, including *P. agglomerans*, *P. ananatis*, *P. stewartii* subsp. *indologenes* and *P. allii*, are causal agents of onion centre rot, a disease which causes considerable losses in onion crops in fields and in storages across several producing regions. While *P. allii* was originally isolated from onion plants and seed [6] and is implicated in postharvest bulb rot [8], it is far less frequently isolated from symptomatic onions compared to the species listed above. A previous study recovered several *P. allii* strains from rainwater [30], and in the present study, we leveraged these isolates together with available plant-associated isolates (primarily from onion) and public genomes. Because these isolates were identified either incidentally or through targeted surveys of onion, the natural distribution of *P. allii* and its broader ecological or host-associated diversity remains poorly understood. Nonetheless, the detection of *P. allii* in rainwater suggests that it may occur in ecological niches distinct from the other onion centre rot pathogens and potentially persist in non-plant reservoirs such as rainwater. Such occurrences raise the possibility of dissemination through the water cycle, as has been suggested for *Pseudomonas syringae* [29], and imply that additional, uncharacterized diversity of *P. allii* may exist in the environmental reservoirs, particularly within components of the water cycle, since sampling of *P. allii* has mostly been limited to onion. Moreover, similar to *P. syringae*, *P. allii* may use its ability to nucleate ice to facilitate its deposition from the atmosphere back to plants [29].

Despite differences in prevalence and isolation sources, onion-pathogenic *Pantoea* species share common virulence strategies centred on the production of phosphonate phytotoxins that induce necrosis in onion tissue, namely, HiVir and Halophos. While HiVir was initially identified on the chromosome of *P. ananatis* [12], it has since been found on plasmids in *P. agglomerans* [14] and *Pantoea vagans* [68]. In contrast, Halophos has not been reported in these species [14,65, 69]. However, both HiVir and Halophos have been identified in strains of *P. stewartii* subsp. *indologenes* [15], and findings from this study indicate that the same is true for *P. allii*. The strains of *P. allii* carrying either the HiVir or Halophos gene clusters induced distinct necrosis symptoms on detached fleshy red onion scales. HiVir+ strains consistently produced white clearing zones around the inoculation point at 4 dpi, whereas Halophos+ strains caused sunken yellow necrotic lesions that were less consistent at the same time point. Nonetheless, the regulatory mechanisms governing these gene clusters and the precise molecular targets of their phytotoxins remain unknown.

Phylogenomic analyses grouped geographically diverse *P. allii* strains into three distinct lineages. Strains in lineages 1 and 2 were distributed across multiple regions, while lineage 3 was composed primarily of strains from the USA. Interestingly, this phylogenetic structure corresponded with the distribution of key virulence factors. Halophos-positive strains were found only in lineages 1 and 2, whereas lineage 3 strains uniformly lacked Halophos but instead carried the HiVir gene cluster. The thiosulfinate tolerance gene cluster (*alt*) was detected exclusively in Halophos-positive strains. In contrast, HiVir-positive strains from lineage 3 lacked *alt*, despite harbouring plasmid B, the plasmid that carries *alt* in Halophos-positive strains (Figs1  3). The factors driving this unexpected overlap between phylogeography and virulence factor distribution are unclear. However, the absence of the *alt* cluster in HiVir-positive *P. ananatis* [70] and *P. agglomerans* [14] has also been observed. Genetically engineered auto-bioluminescent *P. ananatis* strains with a deletion mutation of *alt* was able to induce RSN comparable to that of the wild-type. However, when inoculated at the neck of the onion bulb, the *alt*-deficient strain exhibited a significant loss of bioluminescence in the bulb at 20, 30 and 40 dpi, relative to the wild-type, indicating reduced bacterial colonization [16]. These results suggest that *alt*-lacking strains may be less fit to survive the damage they cause in onion tissue, leading to reduced persistence. Overall, the phylogeographic signal, coupled with the lineage-specific distribution of virulence traits, suggests that the three lineages constitute three separate populations that adapted to three separate ecological niches with limited horizontal gene transfer occurring between them. However, many more questions remain about the evolutionary forces that continue to shape the distribution, ecology and host interactions of *P. allii*. The high number of well-supported sub-clades within lineage 3 reveals that lineage 3 has started to diversify into many separate sub-lineages. The ANI analysis, reflected by the many different LINs at position K (corresponding to an ANI value of 99%), and the gene presence/absence analysis, revealing differences in gene content between sub-lineages, shows that these sub-lineages are possibly adapting to separate ecological niches. Moreover, the fact that strain DOAB1050 does not belong to any of the three lineages suggests that this strain represents a yet under-sampled fourth lineage within *P. allii*.

Two genomic loci encoding T6SS gene clusters were identified in *P. allii* strains. In comparison, up to three T6SS gene clusters have been reported in *P. ananatis* [19,21] and two in *P. agglomerans* [18]. In both species, T6SS-1 encodes a complete and functional antibacterial system that has been experimentally characterized. Based on homology, *P. allii* T6SS-I and T6SS-II correspond to T6SS-1 and T6SS-2, respectively, in *P. ananatis* and *P. agglomerans* [18,19, 21]. Unlike the structurally incomplete T6SS-2 in *P. ananatis* and *P. agglomerans*, *P. allii* strains MAI6007 and BAV3052 harboured computationally predicted complete T6SS-2 (Pall T6SS-II), suggesting potential functional differences. It is also possible that components of the two T6SS clusters in *P. allii* could interact, forming derivatives of the T6SS apparatus. Additionally, the variability of T6SS-II, the presence of plasmid partitioning genes in T6SS-II of DOAB1036 and MAI6022, and the presence of unique phage genes in DOAB1051 (Fig. 4b) suggest that T6SS-II is more prone to recombination and integration events than T6SS-II.

The T6SS gene clusters of *P. allii* are enriched with RHS (rearrangement hot spot) domain-containing proteins, which are often associated with polymorphic C-terminal toxin domains [71,72]. The *vgrG*-1 island of *P. allii* T6SS-I (Fig. 4a) harbours a pentapeptide repeat domain-containing protein (COG1357), a feature also observed in *P. ananatis* DZ-12 [19]. In *P. ananatis*, this protein, identified as the T6SS-secreted effector TseG, interacts with the upstream chaperone TecG (DUF2169) and the downstream immunity protein TsiG (DUF3540). TseG exhibited antibacterial activity against *Escherichia coli* and enhanced virulence in maize, potato and onion plants compared to a *tseG* deletion mutant [19]. Given the high conservation of this toxin-immunity pair across the *Pantoea* genus [19], *P. allii* may employ TseG in a similar functional role. Interestingly, several RHS domain-containing genes in *P. allii* strains DOAB1035 and MAI6007 appear to be pseudogenized, potentially due to the loss of their corresponding immunity genes. Additionally, numerous hypothetical protein-encoding genes found on *vgrG*-2 island may represent orphan immunity genes. While the VgrG effector of *P. agglomerans* pv. betae strain 4188 carries a C-terminal glucosaminidase domain targeting peptidoglycan [18], *P. allii* VgrGs lack this feature, suggesting a distinct mechanism of antibacterial activity to that of *P. agglomerans*. Instead, *P. allii* T6SS may rely on RHS domain-containing effectors such as the pentapeptide repeat domain-containing protein (COG1357) or uncharacterized hypothetical proteins within its *vgrG* islands to mediate bacterial competition and ecological adaptation,

In addition to T6SS genes, *P. allii* harbours Pantailocin islands (Fig. 5) resembling those found in *P. stewartii* subsp. *indologenes* ICMP10132 or *P. ananatis* ATCC35400 [22]. Pantailocins are R-type bacteriocins, bacteriophage-derived protein complexes that resemble contractile phage tails that selectively kill closely related *Pantoea* species [22]. The presence of both types of Pantailocin islands in *P. allii* suggests independent acquisition events rather than inheritance from a common ancestor. Populations of *P. allii* carrying both types of Pantailocin islands may gain a competitive advantage by eliminating Pantailocin-susceptible *P. ananatis* populations. While the production and killing activity of Pantailocins found in *P. allii* remain to be investigated, high synteny and sequence homology of these islands to those in *P. stewartii* subsp. *indologenes* ICMP10132 or *P. ananatis* ATCC35400 suggest similar target specificity. It would also be interesting to determine whether the expanded *P. ananatis*-like Pantailocin island found in DOAB1051 encodes tailocins with altered target specificity.

## Conclusion

This study provided a valuable genomic, phylogenetic and phenotypic characterization of the infrequently isolated *P. allii*, revealing distinct evolutionary lineages and diverse genetic loci relevant to its ecology, pathogenicity and potential as a biocontrol agent. The detection of INA and HiVir gene cluster in strains isolated from environmental reservoirs such as rainwater suggests possible dissemination routes of *P. allii* as an inoculum source for agriculture. While HiVir and Halophos are recognized onion virulence factors, their specific *in planta* targets still remain unknown and require further investigation. The diversity of T6SS-associated toxins and Pantailocin islands points to a complex network of interbacterial interactions that may support both competitive and pathogenic strategies of *P. allii*. Investigating the functional roles of these loci would enhance our understanding of *P. allii*’s persistence in plant-associated environments and their interaction with other microbes.

## Abbreviations

AHL: acyl-homoserine lactone
ANI: average nucleotide identity
BGC: biosynthetic gene cluster
dDDH: digital DNA–DNA hybridization
dpi: days post-inoculation
DUF: domain of unknown function
HSP: high-scoring segment pair
INA: ice nucleation activity
LB: Lysogeny broth
LIN: life identification number
RSN: red onion scale necrosis
T3SS: type III secretion system
T4SS: type IV secretion system
T6SS: type VI secretion system
